# Supplementary figures and images for: Acute Kidney Injury and Chronic Kidney Disease Associated with a Genetic Defect: A Report of Two Cases
Source: Int J Mol Sci. 2025 May 14;26(10):4681. doi: 10.3390/ijms26104681 (PMC12111080; doi:10.3390/ijms26104681)

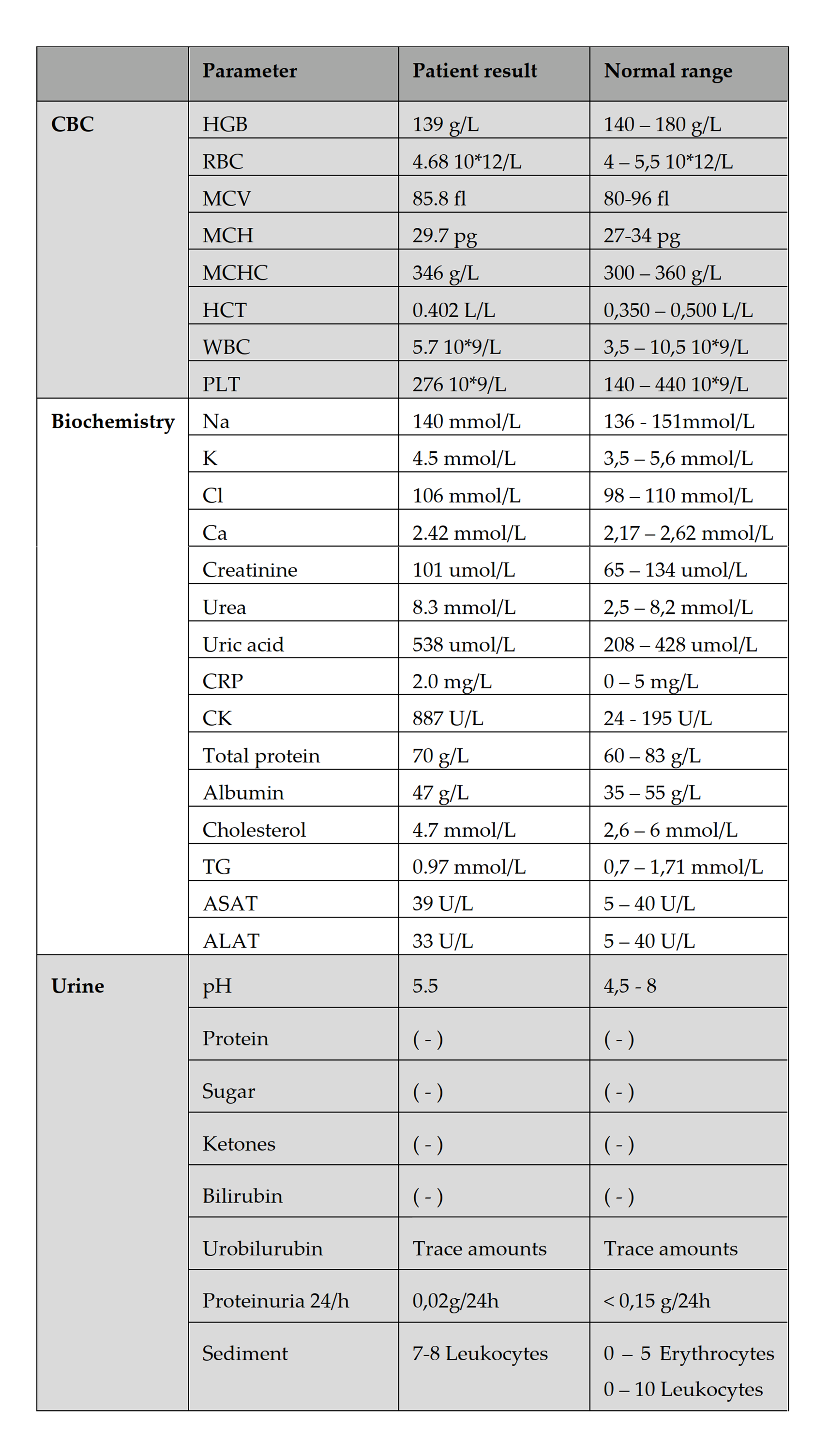

Supplement: Supplementary file 1 [file ijms-26-04681-s001.zip › File S1.png]

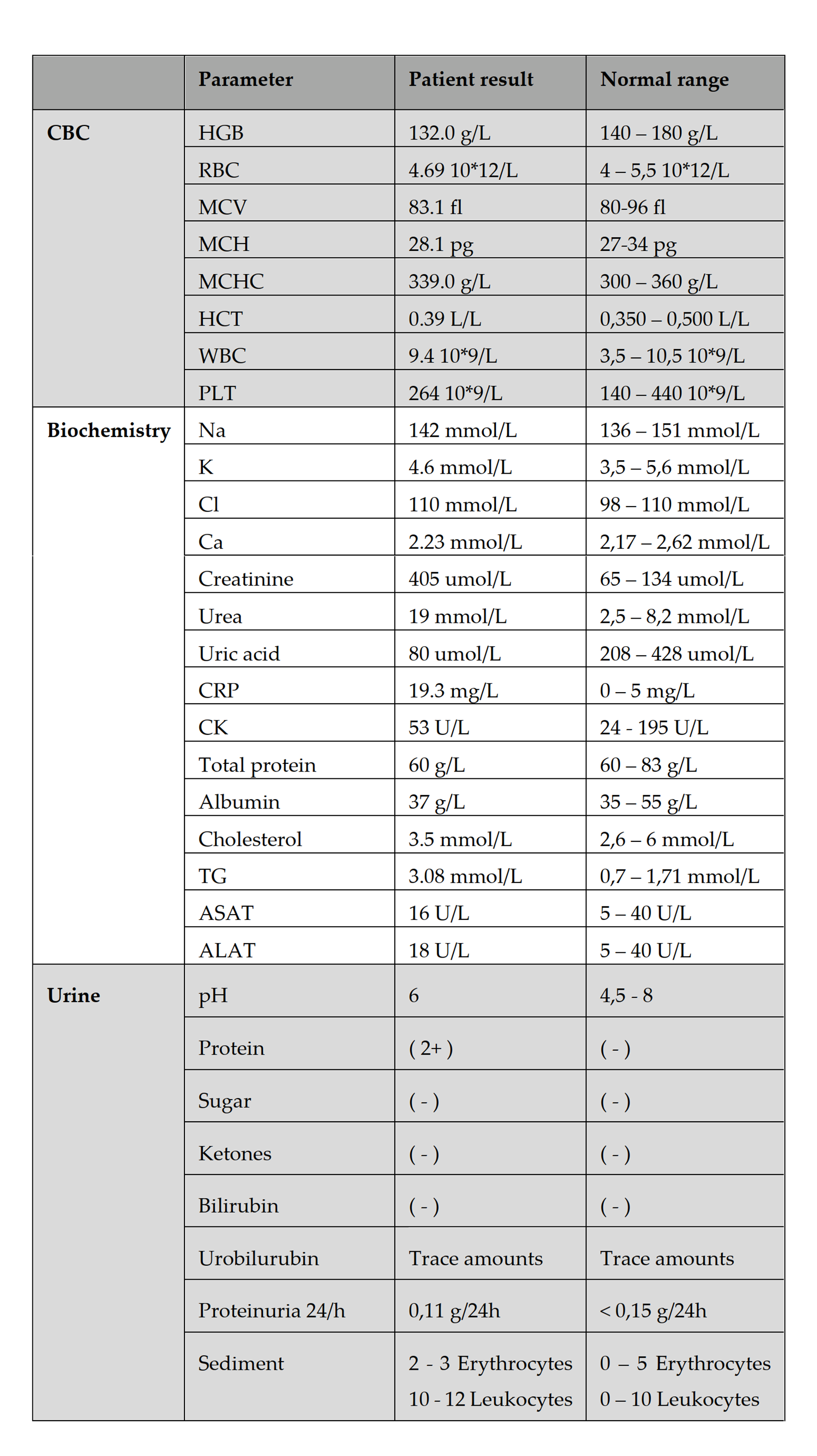

Supplement: Supplementary file 1 [file ijms-26-04681-s001.zip › File S2.png]
